# Protocol for a double-blind randomised placebo-controlled trial of lithium carbonate in patients with amyotrophic Lateral Sclerosis (LiCALS) [Eudract number: 2008-006891-31]

**DOI:** 10.1186/1471-2377-11-111

**Published:** 2011-09-21

**Authors:** Ammar Al-Chalabi, Pamela J Shaw, Carolyn A Young, Karen E Morrison, Caroline Murphy, Marie Thornhill, Joanna Kelly, I Nicholas Steen, P Nigel Leigh

**Affiliations:** 1 MRC Centre for Neurodegeneration Research, King's College London, Department of Clinical Neuroscience, London SE5 8AF, UK; 2 Academic Neurology Unit, Sheffield Institute for Translational Neuroscience (SITraN), University of Sheffield, 385A Glossop Road, Sheffield S10 2HQ, UK; 3 Walton Centre for Neurology & Neurosurgery, Lower Lane, Liverpool L9 7LJ, UK; 4 College of Medical and Dental Sciences, University of Birmingham, Birmingham, B15 2TT, UK; 5 Mental Health and Neuroscience Clinical Trials Unit, King's College London, Institute of Psychiatry, London SE5 8AF, UK; 6 Institute of Health and Society, University of Newcastle upon Tyne, 21 Claremont Place, Newcastle upon Tyne, NE2 4AA, UK; 7 Brighton and Sussex Medical School, Trafford Centre for Biomedical Research, University of Sussex, Falmer, East Sussex BN1 9RY, UK

## Abstract

**Background:**

Amyotrophic lateral sclerosis is a rapidly progressive neurodegenerative disorder characterised by loss of motor neurons leading to severe weakness and death from respiratory failure within 3-5 years. Riluzole prolongs survival in ALS. A published report has suggested a dramatic effect of lithium carbonate on survival. 44 patients were studied, with 16 randomly selected to take LiCO_3 _and riluzole and 28 allocated to take riluzole alone. In the group treated with lithium, no patients had died (i.e., 100% survival) at the end of the study (15 months from entry), compared to 71% surviving in the riluzole-only group. Although the trial can be criticised on several grounds, there is a substantial rationale from other laboratory studies that lithium is worth investigating therapeutically in amyotrophic lateral sclerosis.

**Methods/Design:**

LiCALS is a multi-centre double-blind randomised parallel group controlled trial of the efficacy, safety, and tolerability of lithium carbonate (LiCO_3_) at doses to achieve stable 'therapeutic' plasma levels (0.4-0.8 mmol/L), plus standard treatment, versus matched placebo plus standard treatment, in patients with amyotrophic lateral sclerosis. The study will be based in the UK, in partnership with the MND Association and DeNDRoN (the Dementias and Neurodegnerative Diseases Clinical Research Network). 220 patients will be recruited. All patients will be on the standard treatment for ALS of riluzole 100 mg daily. The primary outcome measure will be death from any cause at 18 months defined from the date of randomisation. Secondary outcome measures will be changes in three functional rating scales, the ALS Functional Rating Scale-Revised, The EuroQOL (EQ-5D), and the Hospital Anxiety and Depression Scale.

Eligible patients will have El Escorial Possible, Laboratory-supported Probable, Probable or Definite amyotrophic lateral sclerosis with disease duration between 6 months and 36 months (inclusive), vital capacity ≥ 60% of predicted within 1 month prior to randomisation and age at least18 years.

**Discussion:**

Patient recruitment began in June 2009 and the last patient is expected to complete the trial protocol in November 2011.

**Trial registration:**

Current controlled trials ISRCTN83178718

## Background

Amyotrophic lateral sclerosis (ALS; motor neuron disease, MND) is a rapidly progressive neurodegenerative disorder characterised by loss of corticospinal tract motor neurons and spinal anterior horn motor neurons leading to severe weakness and death from respiratory failure usually within 3-5 years [1]. In about 5% of cases there is a family history of ALS, and in about a quarter of such cases, mutation in the SOD1, TARDBP, FUS or OPTN genes is responsible. ALS is the first neurodegenerative disease to provide hope that neuroprotective strategies may be feasible. Riluzole prolongs survival in ALS with a reduction in mortality of ~7% at 18 months for 100 mg daily riluzole versus placebo and a relative risk reduction of ~35% [2-4]. Riluzole has been licensed by the MHRA and approved by NICE http://www.nice.org.uk. Nonetheless there remains a pressing need for effective treatments that further slow disease progression, while maintaining quality of life.

Any drug that can be shown to slow the course of ALS in a clinically significant way and to be safe and well tolerated will be an important advance for patients with this disease. A published report has suggested a dramatic effect of lithium carbonate on survival, albeit in a very small group of patients [5]. 44 patients were studied, with 16 randomly selected to take LiCO3 and riluzole and 28 allocated to take riluzole alone. In the group treated with lithium, no patients had died (i.e., 100% survival) at the end of the study (15 months from entry), compared to 71% surviving in the riluzole-only group. The difference in survival was significant at 12 and 15 months. There were also significant differences in the rate of functional deterioration measured by MRC Manual Muscle Testing scores (MMT; 18% decline in LiCO_3 _+ riluzole group versus 35% in the riluzole only group), the Norris scale (11% versus 46%), ALSFRS-R (14% versus 40%), and forced vital capacity (FVC; 14% versus 33%).

Unfortunately, the trial design can be criticised on several counts. First, the patients were not well matched for age, the LiCO_3 _group being approximately three years younger at age of onset than the control (riluzole only) group, which is known to be a prognostic factor. Secondly, and crucially, the groups were not truly randomised, but 'randomly selected'- no details of how this was done were provided, although the authors stated that patients were selected on the basis of matched forced vital capacity and pattern of clinical presentation. The study was single-blind, with the physicians carrying out the functional assessments being blinded. Adjustments of plasma lithium levels were carried out by an unblinded observer, and the patients were not blinded. The dose of lithium carbonate ranged from 300 mg to 450 mg daily, with plasma lithium levels targeted to reach 0.4-0.8 mmol/l.

ALS is a heterogeneous disease, and each group of patients entering a trial will differ, to a greater or lesser extent, in the average rate of progression measured by death or by measures of function such as the ALSFRS-R. The smaller the sample, the greater is the chance that there will be major differences in average rates of progression between treatment groups. While large samples and true randomisation will usually balance differences in prognostic factors such as the anticipated rate of disease progression between patients taking the active drug or placebo, this is not always the case [6,7]. Even in large randomised trials in which patients are recruited using standardized entry criteria, there are patients with slow disease progression as well as patients with a high risk of dying before the end of the study [3,4,6]. It is therefore possible, by chance, to select a small group of patients with unusually good prospects for survival. On the other hand, despite the weaknesses of trial design, the size of the observed difference between the lithium and control groups (i.e., the assumed drug effect) is difficult to explain on the basis of imbalance of major prognostic factors alone.

The same study [5] also included a series of experiments in mice transgenic for the G93A Superoxide Dismutase 1 (SOD1) mutation, known to cause ALS in humans. There was a prolonged mean survival time (p < 0.001) and increased disease duration (p = 0.05) with lithium treatment compared to saline treatment, starting at 75 days of age. The mean survival time in the lithium group was 148 ± 4.3 days (n = 20), compared to 110.8 ± 5 days (n = 20) in vehicle treated mice, representing ~36% increase in lifespan. Disease duration was increased from a mean of 9 days to more than 38 days (i.e., > 300%) in lithium compared to vehicle treated mice.

The authors comment that even when lithium treatment was started at the onset of motor symptoms there was still a comparable increase in disease duration, but these data were not shown. In addition, lithium delayed the onset of paralysis and limb adduction and also improved function on tests such as rotarod, grip strength and stride length. The authors found that lithium treatment increased the number of surviving α motor neurons in the G93A mice and had a number of other effects on pathological changes including increasing the numbers of Renshaw-like neurons in the spinal cord. They hypothesised that this was mediated through an effect on neurogenesis, since lithium has been reported to produce neurogenesis in physiological conditions in the hippocampus, albeit not in the spinal cord. They also reported that lithium rescues spinal cord mitochondria and facilitates the clearance of α-synuclein, ubiquitin and SOD1, and that it increases the number of autophagic vacuoles in the spinal cord.

There is a substantial rationale from other laboratory studies that lithium is worth investigating therapeutically in ALS. One previous study also identified a modest protective effect of lithium in the ALS transgenic mouse [8]. Lithium has been shown to have neuroprotective effects in several models of neurodegeneration through a variety of mechanisms [9,10]. The kinesin light chain, a key component of the cellular motor mediating fast anterograde axonal transport, is a substrate for glycogen synthase kinase 3 Beta (GSK3β) [11-13] and lithium reverses deficits in axonal transport in a microtubule-associated protein tau (MAPT) over-expression model in drosophila [14]. Fast axonal transport of mitochondria is impaired by SOD1 mutations, resulting in a maldistribution of mitochondria in the neuron [11] so lithium might counteract this.

The neuroprotective effects of lithium are thought to be mediated to some degree and in some models through GSK3β inhibition [9,10,15,16] but also by modification of NMDA-subtype glutamate receptor function through inhibition of NR2B tyrosine phosphorylation [17], activation of cell survival factors such as the PI 3- kinase/Akt signalling pathway, [1] the induction of neurotrophic proteins, including brain-derived neurotrophic factors (BDNF) [9,18,19] and up-regulation of heat-shock protein and Bcl-2 [9,20-23]. Lithium enhances neurogenesis in various experimental systems [5,24]. In addition, lithium may promote autophagy through the inhibition of inositol-monophosphatase-15, and thus through the neuroprotective effects of autophagy in neurodegeneration [25-28]. However, the effects of lithium on autophagy are complex, as lithium may reduce autophagy by inhibiting GSK3β, but stimulate it by inducing the mammalian target of rapamycin (mTOR)-dependent autophagy [26]. Thus combinations of lithium with agents that inhibit mTOR may prove more useful in neuroprotection than lithium alone. We have no data on possible interactions between lithium and riluzole.

Thus, despite reservations regarding the strength of the clinical trial data, there is a strong argument to carry out a phase II/III study of LiCO_3 _in ALS. We take the view that such a study should be powered to detect a large effect, i.e., close to that observed in the original human study [5].

There are various systematic reviews of neuroprotective strategies in ALS, as well as Cochrane systematic reviews of riluzole and failed treatments [29]. In essence, apart from Riluzole, none of the many agents tested have had any positive effect on survival or function in ALS, and there remains a pressing need for disease-modifying therapy, the aim being to slow disease progression while maintaining reasonable quality of life, in conjunction with symptomatic treatments and palliative care.

It is hoped that results from our study will be used to inform decisions of whether lithium should be added to riluzole in the routine management of patients with ALS, to improve their survival, motor function and quality of life without significant adverse effects.

The protocol includes dose titration and monitoring to achieve therapeutic plasma levels of > = 0.4- < = 0.8 mmol/L.

Lithium has been prescribed in long term use over many decades for other patient groups [30]. Because the effective dose is fairly close to the toxic dose, it is necessary to conduct careful dose titration [30-32]. We have taken advice on initiation and monitoring of therapy from senior psychiatrists with extensive expertise in the pharmacology and clinical use of lithium therapy and will follow the NICE guidelines (http://www.nice.org.uk, Guideline No 38, 2006). The protocol will also involve un-blinded "safety monitoring" physicians who will be responsible for monitoring lithium levels and assessing patients for all aspects of safety and possible toxicity.

## Methods/Design

For further detail please see Additional File 1.

### Primary Objective

To determine whether lithium carbonate (LiCO_3_), in doses achieving blood levels of 0.4-0.8 mmols/L, combined with standard ALS treatment, significantly prolongs survival in ALS over 18 months, compared with standard treatment alone.

### Secondary Objectives

To monitor the safety of treatment with LiCO_3 _over 18 months.

To determine whether treatment with LiCO_3 _slows the rate of functional deterioration over 18 months.

To determine whether treatment with LiCO_3 _affects quality of life (QoL) or mental state (anxiety, depression) in patients with ALS, over 18 months

### Study Design

LiCALS is a multi-centre (UK) double-blind randomised parallel group controlled trial of the efficacy, safety, and tolerability of lithium carbonate (LiCO_3_) at doses to achieve stable 'therapeutic' plasma levels (0.4-0.8 mmol/L), plus standard treatment, versus matched placebo plus standard treatment, in patients with ALS. The study will be based in the UK, in partnership with the MND Association and DeNDRoN.

A total of 220 patients will be recruited. All patients will be on the standard treatment for ALS (riluzole 100 mg daily) as per routine clinical practice. The study will evaluate the efficacy and safety of lithium carbonate given orally, combined with standard treatment, compared to matched placebo with standard treatment. All participants involved will be required to be taking a stable dose of riluzole 50 mg orally twice daily for at least 28 days (four weeks) prior to screening. All participants will be consented in accordance with the declaration of Helsinki. The study has been approved by the South East Research Ethics Committee (reference 09/H1102/15). Following consent, participants will be randomised 1:1 to either LiCO_3 _plus standard treatment or matched placebo plus standard treatment.

### Primary outcome measure

Death from any cause at 18 months defined from the date of randomisation.

Death will be assessed by 3 monthly (telephone) status reports and by standard clinic reports and confirmed by written evidence from the trial staff or general practitioner, and a death certificate will be obtained for all deaths. Further ascertainment of status will be sought from the NHS Information Centre tracing service if required, to avoid loss to follow-up of this primary outcome. Previous trials obtained 100% ascertainment of death through death certificates (i.e., obtained for every death) and documented survival at cut-off for surviving patients.

### Secondary outcome measures

#### Slope of ALS Functional Rating Scale-Revised (ALSFRS-R) scores

[32,33]

Functional measures (ALSFRS-R) will be assessed at visits by blinded trained trial staff, according to standard operating procedures (SOPs).

#### Change in EuroQOL (EQ-5D)

A standardised instrument for use as a measure of health outcome, describing states of health in five dimensions: mobility, self-care, usual activities, pain or discomfort and anxiety or depression [34,35].

#### Change in Hospital Anxiety and Depression Scale (HADS)

A 14 item questionnaire, designed to detect the presence and severity of mild degrees of mood disorder, anxiety and depression [36].

The ALSFRS-R [32,37] and EQ-5D [38] are validated for telephone use.

#### Definition of end of study

The end of the study will be the last participant's final study contact, at final visit (month 18/week 77).

SAEs will be monitored for a further 30 days after stopping the trial treatment, or until resolution.

The open label extension study will commence when the first patient completes Month 18 (week 77) and will run in parallel with the double blind study until the completion of the double blind primary analysis.

#### Subject Population

##### Inclusion criteria

Patients with Possible, Laboratory-supported Probable, Probable or Definite ALS according to the revised version of the El Escorial World Federation of Neurology criteria (The 'Airlie House Statement': http://www.wfnals.org)

These criteria are internationally accepted research diagnostic criteria with high specificity and sensitivity. The onset form (bulbar or limb) and disease type (familial or sporadic) will be recorded; source documents will include a full report of an electromyogram (EMG) reported by an experienced neurophysiologist as compatible with ALS. The neurological exam should be performed by a physician.

Disease duration ≥ 6 months and ≤ 36 calendar months (inclusive), with disease onset defined as date of first muscle weakness, or dysarthria. SVC ≥ 60% of predicted within 1 month prior to randomisation

Age: ≥ 18 years (inclusive). In the case of a female with childbearing potential, the patient must not be pregnant or breast-feeding. Women of childbearing potential will have a urine pregnancy test before randomisation; and at each clinic visit. The results of those must be negative. Women of childbearing potential should use adequate contraception. Patients should be continuously treated with riluzole for at least 4 weeks prior to screening (28 days inclusive) and stabilised at 100 mg/day (50 mg bid) without significant adverse drug reactions. Trial participants should be capable of understanding the information given and giving fully informed consent prior to any study specific procedures.

##### Exclusion criteria

Participation in another therapeutic study within the preceding 12 weeks or use of other investigational drugs or agents. Tracheostomy, or assisted ventilation of any type during the preceding three months. Existing gastrostomy, unless elective and not currently used for nutritional support or hydration. Any medical condition known to have an association with motor neuron dysfunction which might confound or obscure the diagnosis of ALS. Presence of any concomitant life-threatening disease or any disease or impairment likely to interfere with functional assessment. Confirmed hepatic insufficiency or abnormal liver function (ALT greater than 1.5 times the upper limit of the normal range) within one month of randomisation. That blood test may be repeated in the case of initial abnormal results; if the levels return to normal, the patient may then be included in the study. Renal insufficiency (serum creatinine ≥ ULN for the centre/local laboratory) within one month of randomisation. Recorded diagnosis or evidence of major psychiatric disorder or clinically evident dementia. Known allergy or hypersensitivity to lithium, or its excipients. Likely to be uncooperative or to fail to comply with the trial requirements or to be inaccessible in the event of an emergency. Subjects with significant haematological, biochemical and autoimmune abnormalities, as judged by the study physician. If a woman of childbearing potential, unable or unwilling to use effective contraception during the study. Patients with active inflammation/infection at screening or Baseline (Day 0). Patients presenting with active inflammation/infection can be reassessed at a later date, and included in the trial if the infection/inflammation has cleared. Patients already taking lithium in any form. Presence of a medical condition contra-indicative to the use of lithium, according to the BNF http://www.bnf.org/bnf/

#### Screening, Recruitment and Consent

Patients will be identified through clinics at each of the 10 centres, which are all regional MND Care Centres. They will be given the patient information sheet to read, and will have 7-14 days to decide whether to participate in the study. At clinic, there will be opportunity for the participants to ask questions of a member of staff trained in all trial procedures, as delegated by the PI. The Principal Investigator at each site will ensure that the participants meet the inclusion and exclusion criteria at the point of screening.

After signing the consent form, participants will have blood tests to measure their thyroid function, detect any renal and hepatic insufficiency, any haematological, biochemical or immunological abnormalities, and their SVC will be measured. If they are a woman of childbearing potential, they will have a urine pregnancy test at each of their clinic visits. Those procedures should be done within one month prior to randomisation.

A screening log will be kept at site to document details of patients invited to participate in the study. For patients who decline or are ineligible, this will document any reasons available for non-participation (where provided). The log will ensure potential participants are only approached once.

The original signed consent form will be retained in the Investigator Site File, with a copy in the participant's hospital medical notes, and a copy provided to the participant. The participant will specifically consent to their GP being informed of their participation in the study.

The right to refuse to participate without giving reasons will be respected.

#### Study Medication

##### Description of Randomised Treatments

###### Placebo

Matching placebo tablets will be manufactured for the 295 mg lithium carbonate tablets. These will be identical in appearance to the active tablets-white tablets of tablets of approximately 1.1 cm in diameter, with a break notch on one side.

###### Lithium Carbonate

The experimental intervention is lithium carbonate, in combination with normal standard treatment for ALS (riluzole 50 mg bd). Lithium carbonate is licensed for the treatment and prophylaxis of mania, bipolar disorder (manic-depressive disorder), recurrent depression, and the control of aggressive or self-mutilating behaviour. Within the context of this study, lithium carbonate will be used in a new target population.

Reported side effects of lithium carbonate are given later.

##### Selection of Doses for the Trial

The study medication is being used within normal clinical doses. The medication will be titrated to achieve a blood plasma level of ≥ 0.4- ≤ 0.8 mmol/l.

##### Selection & Timing of Dose for Each Participant

Interventions will be available in 295 mg tablets of lithium carbonate or matching placebo.

Tablets will be taken once daily, starting with one tablet (295 mg daily) initially, titrated up to two or three tablets daily over the first four weeks of treatment, depending on blood lithium levels. Blood levels will be measured at baseline, 7 days (12 hours +/-30 minutes after previous evening dose), and at 14 days (12 hours +/-30 minutes after previous evening dose). The target range for the lithium plasma level will be between ≥ 0.4 mmol/l and ≤ 0.8 mmol/l. Further plasma level measurements will be taken at 21 and 28 days to confirm the target range has been achieved. Ongoing lithium level monitoring will be scheduled for week 8 and week 12, and 3 monthly thereafter for the duration of the study.

Dose adjustments can be made by reducing back to 2 tablets daily or to 1 tablet daily as required. It is anticipated that most patients will remain on 2 tablets throughout the duration of the trial.

Sham dose adjustments will also be made to patients on placebo to maintain blinding in clinical sites as per SOP.

##### Blinding of Investigational Medicinal Product

Active study medication (Lithium carbonate) and placebo will be identical.

##### Identity & Supply of Investigational Medicinal Product

Lithium carbonate-Apogepha (Germany)

The trial medication is supplied as white tablets of approximately 1.1 cm in diameter. There is a break notch on one side of the tablets.

##### Packaging & Labelling of Investigational Medicinal Product

Active study medication and placebo will be supplied in blisters of 21 tablets. For months 1, 2 and 3, five blisters will be packaged into 1 month kits. 3 of these kits, each bearing the same kit number, will be packaged together and dispensed directly to the study site.

Thereafter, 14 individual blisters will be supplied in 3-monthly patient kits for months 4-6, 7-9, 10-12, 13-15 and 16-18. Each of those kits will have a different kit number. Packaging and labelling will be completed in accordance with Good Manufacturing Practice (GMP) Annex 13 requirements and GCP, by Haupt Pharma, Brackenheim, Germany.

##### Prescription of Investigational Medicinal Product

Study medication will be prescribed by an authorised study physician according to the protocol, using a trial specific prescription. Medication will be dispensed according to local pharmacy practice.

Participants will be informed of potential adverse reactions and advised to seek medical help and contact the research team, if required. Patients will carry cards with an emergency 24 hour code break number (Guy's Medical Toxicology Unit, (0)207 358 9167), which will also be printed on the study medication packs.

Documentation of prescribing, dispensing and return of study medication shall be maintained for study records in the pharmacy file and reconciled with the investigator site file at the end of the study.

##### Dispensing & Distribution of Investigational Medicinal Product

Study drug will be stored in a secure area with limited access within each local pharmacy according to the storage requirements documented on the clinical trial label (store in the original package and protected from moisture), prior to dispensing for each participant's baseline visit. A temperature log will be maintained as per local pharmacy procedures.

Study medication will be distributed to the 10 study site pharmacies by and from Haupt Pharma, Brackenheim, Germany. Study medication receipt will be recorded in the study pharmacy file. A study medication dispensing and return log will be maintained by the site pharmacies.

Supplies of the study medications will be dispensed to the patient at Months 0, 1, 2, 3, 6, 9, 12 and 15.

##### Administration of Investigational Medicinal Product

Tablets will be taken by participants using their normal methods for medicines management in a single dose at night. Participants will be provided with written information from the doctor detailing the dose to be taken.

Following stabilisation of blood levels over the first 3 months, plasma level monitoring can be measured every 3 months, except in the following circumstances, when monitoring needs to be done more frequently: presence of intercurrent illness, symptoms or signs of lithium toxicity, or if the patient is taking interacting drugs e.g. non-steroidal anti-inflammatory agents, diuretics, steroids, metronidazole, tetracycline, angiotensin converting enzyme (ACE) inhibitors; or if there is a change in fluid or sodium intake. If the study medication is given crushed via a feeding tube, as opposed to as whole tablets, monitoring of Li Levels will also need to be more frequent. All medication will be monitored by a lithium physician (unblinded).

#### Unused Trial Study Medication & Study Medication Accountability

At the end of each visit (months 1, 2, 3, 6, 9, 12,15, and 18) participants will be asked to return any surplus study drug in the original packaging to the research team, who will verify and document compliance and discuss any discrepancies immediately with the participant. All unused study medication and packaging will then be sent to the local pharmacy where the study monitor will check and arrange collection of returns. Monitoring checks will be carried out between the eCRF and pharmacy to ensure the number of tablets returned is correctly documented.

##### Concomitant Medications

All concomitant drug therapies received will be recorded at baseline and follow-up assessment.

Contraindications include hypersensitivity to lithium or to any of the excipients.

Lithium can cause neurotoxicity, which can happen without an increase in blood lithium levels. Neurotoxicity usually follows interaction with medications; drugs implicated include selective serotonin reuptake inhibitors (SSRIs), calcium channel blockers, carbamazepine, phenytoin, neuroleptics and methyldopa. The protocol will therefore involve a lithium physician (unblinded) who will be responsible for monitoring lithium levels and making dose adjustments as described later. The treating physician (blinded) will assess patients, review adverse events, and consider possible toxicity. Details of all other agents that might interact with lithium can be found in the British National Formulary (BNF) http://www.bnf.org/bnf/.

The treating physician (blinded) will be provided with an emergency 24 hr unblinding contact number (Guy's Medical Toxicology Unit, 0207 358 9167).

Participants should not receive any other investigational drugs or agents during their participation in the study.

##### Safety Monitoring

The following blood tests will be done at regular intervals throughout the study, as indicated in Table 1.

**Table 1:** Safety Blood Tests

Phase	Pre-Randomisation	Weekly visits						Monthly visits						W/l
Blood test	Wk -4 to Day 0	0	1	2	3	4	8	3	6	9	12	15	18	Withdrawal
Haematology	x					x	x	x	x	x	x	x	x	
Biochemistry	x		x	x	x	x	x	x	x	x	x	x	x	
Thyroid Function	x								x		x		x	

Li Levels	x	x	x	x	x	x	x	x	x	x	x	x	x	x

Routine haematology: white blood cell count and differential (eosinophils, basophils, neutrophils, lymphocytes and monocytes), red blood cell count and indices (PCV, MCV, MCH, MCHC), haemoglobin, platelets; biochemistry: (including liver and renal function), alkaline phosphatase, bilirubin, albumin, sodium, potassium, calcium, corrected calcium, phosphate, total protein, globulin, urea, serum creatinine, ALT. Thyroid function will be assessed by measurement of thyroid stimulating hormone.

Haematology, biochemistry, thyroid function and lithium levels can be done within 28 days pre-randomisation or at baseline. All results must be available prior to the patient being randomised.

##### Method of Identification of Patients

Patient identification number will be allocated by registering the patient on the MACRO eCRF system, after consent has been signed. The system will generate a unique identifier to be used throughout the study.

The PIN will be a five digit number; the initial two digits indicate the centre

(Birmingham = 01; King's College Hospital = 02; Liverpool = 03; Manchester = 04; National Hospital for Neurology and Neurosurgery = 05; Newcastle = 06; Oxford = 07; Plymouth = 08; Preston = 09; Sheffield = 10), and then a three-digit number indicating the number within the centre.

##### Method of Randomisation

Patients will be allocated to placebo or lithium carbonate (ratio 1:1) by sites via an online system based at the Mental Health & Neuroscience Clinical Trials Unit (MH&N CTU) based at the Institute of Psychiatry. The randomisation website address is https://ctu.iop.kcl.ac.uk/randomisations/login.asp

Allocation will be stratified by centre and site of disease onset, by stratified block randomisation with randomly varying block sizes.

Only site staff authorised to request randomisation will be sent passwords for the randomisation system. Requests for passwords are via the trial manager to the MH&N CTU.

##### Implementation Procedures

Blinded packs of study medications will be sent to the site pharmacies.

Once an eligible patient has completed the baseline assessment and provided written informed consent, online randomisation will be requested by site. Emails confirming the treatment kit allocation will automatically be generated to the requestor, the site PI, the site pharmacist and the trial manager. A study specific prescription will be completed and sent to pharmacy for dispensing. The confirmation e mail will be attached to the study prescription, and cross-checked against it, at the point of dispensing.

For baseline, week 4 and week 8 visits, this initial treatment kit number will be used for prescribing study medication.

For months 3, 6, 9, 12 and 15, the online system must be accessed again and a new kit number allocated per patient per visit.

Each kit will correspond to the appropriate study arm the patient was randomised to receive.

Any problems with the online randomisation system should be reported to the trial manager or to the MH&N CTU at the email address below:

Contact details for Randomisation: Randomization_request@iop.kcl.ac.uk.

##### Blinding

Assignment to either lithium carbonate or placebo will be blinded to the participants, investigators and pharmacy (double-blind). Blinding of patients and treating physicians to allocation status will be assured by identical tablet appearance, by identical labelling between placebo and doses of active drug, and by sham dosage adjustments made by a lithium physician (unblinded) in the control group to mirror titration of lithium dosage in the intervention group. For safety reasons, lithium physicians (unblinded) will monitor plasma lithium levels, safety bloods, and symptoms. Every effort will be made to maintain strict blinding, although for practical reasons, the unblinded lithium physicians will be part of the same unit as the treating physicians (blinded).

A set of sealed code-break envelopes will be prepared by Haupt Pharma and sent to Guy's Medical Toxicology Unit; these envelopes should be opened only where knowledge of the randomised treatment is needed to optimise the clinical management of the patient. Code breaks will not be routinely opened for participants who complete study treatment.

A sealed randomisation list will also be held by MH&N CTU as back-up.

If a request for code break is received from a physician (e.g. the patient's general practitioner) outside the research team, Guy's Medical Toxicology Unit will attempt to contact the research team to verify the request before the code is broken.

If the code is broken, details including patient study number, the date code break was performed, the person who broke the code, and reason for code break shall be recorded on a record card within the code break envelope and maintained. If clinically indicated, the participant will be withdrawn from study medication.

Accidental unblindings will be dealt with on a case by case basis, if and when they arise. We will continue to collect the patient's data according to the visit schedule, unless the patient withdraws their consent.

### Study Data

#### Trial Database

An electronic Case Report Forms (eCRF) will be created using the InferMed Macro system. This system is regulatory compliant (GCP, 21CRF11, EC Clinical Trial Directive). The eCRF will be created in collaboration with the trial statisticians and the investigators and maintained by the MH&N Clinical Trials Unit. It will be hosted on a dedicated secure server within KCL.

Source data will be entered by authorised staff onto eCRF with a full audit trail.

#### Trial Database Website Address

http://www.ctu.co.uk (click 'studies' tab)

#### Database passwords

Database access will be strictly restricted through passwords to the authorised research team. The trial manager will request usernames and passwords from the MH&N CTU. It is a legal requirement that passwords to the eCRF are not shared, and that only those authorised to access the system are allowed to do so. If new staff members join the study, a personalised username and password should be requested via the Trial Manager.

#### Data Handling & Confidentiality/Format of Records

Data will be handled, computerised and stored in accordance with the Data Protection Act, 1998. Participants will be identified on the study database using a unique code and initials. The investigator will maintain accurate patient records/results detailing observations on each patient enrolled.

#### Identifiable Data

All participant contact/screening and recruitment data will be will be stored centrally within an Access database which will have restricted access from password protected computers, in order to facilitate NHS Information Centre monitoring and collection of death certificates. This database will be destroyed at study end. Accrual data uploaded to the UKCRN portfolio database will be anonymised and collated by the Trial Manager to DeNDRoN.

##### Main Database

SAE data will be collected on paper case report forms (pCRF) and faxed to the MH&N CTU. SAEs will be transcribed to eCRF. For all other data collected there will be a mixture of paper and electronic source documents (to be outlined in the Data Management SOP) that will be entered onto the main database. The Principal Investigator will provide an electronic signature for each patient Case Record Form once all queries are resolved and immediately prior to database lock, when instructed to do so by the Trial Manager. The electronic signoff page will not be available to sites until this time.

##### Lithium MACRO Database

A separate database will hold details linked to lithium monitoring in which lithium physicians (unblinded) have 24 hrs after being made aware of results to enter data.

At the end of the study, essential documentation will be archived in accordance with sponsor and local requirements. The retention of study data will be the responsibility of the Chief Investigator.

#### On-Site/Central Monitoring

The Trial Manager will conduct on-site/central monitoring. The Data Manager/Statistician may identify data fields that should be checked against the source data during site monitoring visits, the specifics will be outlined in a Data Management SOP. Where there are data queries the research nurses will be responsible for resolving the queries. The Trial Manager will review responses before closing the query.

#### Assessments/Data Collection

Written informed consent must be obtained prior to screening and any other study specific procedures taking place.

Proposed frequency and duration of follow-up (Table 2) is based on considerations for dose adjustment and safety monitoring, as suggested in the NICE guidelines on lithium: http://www.nice.org.uk; Guidelines No 38. All visit dates are calculated from the date of the randomisation visit (Day 0). Study procedures are listed in Table 3.

**Table 2:** Visit Schedule

Pre-Randomisation	Week 3/Day 21 (+/- 3 days)	Week 12 (+/- 7 days)	Month 12 (+/- 7 days)
A detailed list of drugs and other agents that might interact with lithium will be provided to volunteers and to their general practitioners, together with a list of 24 hour emergency contact numbers.	Dose monitoring (Li levels)	ALS Interventions	ALS Interventions
Informed consent	Biochemistry	ALSFRS-R	ALSFRS-R
Registration/Demographics	Urine Pregnancy Test Adverse Events	EQ-5D	EQ-5D
SVC	Concomitant medications	HADS	HADS
ALS History		Physical Exam (inc vital signs)	Physical Exam (inc vital signs)
ALS Interventions		Dose monitoring (Li levels)	ECG
Medical HistoryNeurological Exam		Haematology	Dose monitoring (Li levels)
Inclusion/Exclusion Criteria Physical Exam (inc vital signs)		Biochemistry	Haematology
ALSFRS-R		Urine Pregnancy Test Adverse Events	Biochemistry
EQ-5D		Concomitant medications	TFTs
HADS		Trial Medication Log	Urine Pregnancy Test Adverse Events
ECG		Drug Dispensing & Returns	Concomitant medications
Dose monitoring (Li levels)			Trial Medication Log
Haematology			Drug Dispensing & Returns
Biochemistry			
Thyroid Function Tests			
Urine Pregnancy Test			
Concomitant Medications			
Adverse Events			
Drug Dispensing			
Trial Medication Log			

**Week 1/Day 7 (+/- 3 days)**	**Week 4/Day 28 (+/- 3 days)**	**Month 6 (+/- 7 days)**	**Month 15 (+/- 7 days)**

Dose monitoring (Li levels)	Dose monitoring (Li levels)	ALS Interventions	ALS Interventions
Biochemistry	Haematology	ALSFRS-R	ALSFRS-R
Urine Pregnancy Test	Biochemistry	EQ-5D	EQ-5D
Adverse Events	Urine Pregnancy Test Adverse Events	HADS	HADS
Concomitant medications	Concomitant medications	Physical Exam (inc vital signs)	Physical Exam (inc vital signs)
	Trial Medication Log	ECG	Dose monitoring (Li levels)
	Drug Dispensing & Returns	Dose monitoring (Li levels)	Haematology
		Haematology	Biochemistry
		Biochemistry	Urine Pregnancy Test Adverse Events
		TFTs	Concomitant medications
		Urine Pregnancy Test Adverse Events	Trial Medication Log
		Concomitant medications	Drug Dispensing & Returns
		Trial Medication Log	
		Drug Dispensing & Returns	

**Week 2/Day 14 (+/- 3 days)**	**Week 8 (+/- 3 days)**	**Month 9 (+/- 7 days)**	**Month 18/Week 77 (+ 7 days)**

Dose monitoring (Li levels)	Dose monitoring (Li levels)	ALS Interventions	ALS Interventions
Biochemistry	Haematology	ALSFRS-R	ALSFRS-R
Urine Pregnancy Test Adverse Events	Biochemistry	EQ-5D	EQ-5D
Concomitant medications	Urine Pregnancy Test Adverse Events	HADS	HADS
	Concomitant medications	Physical Exam (inc vital signs)	Physical Exam (inc vital signs)
	Trial Medication Log	Dose monitoring (Li levels)	ECG
	Drug Dispensing & Returns	Haematology	Dose monitoring (Li levels)
		Biochemistry	Haematology
		Urine Pregnancy Test Adverse Events	Biochemistry
		Concomitant medications	TFT's
		Trial Medication Log	Urine Pregnancy Test Adverse Events
		Drug Dispensing & Returns	Concomitant medications
			Trial Medication Log
			Drug Returns
			Unscheduled Visits

**Table 3:** Table of events-summary of study procedures

Phase	Pre-Randomisation	Double-Blind Treatment												
**Study Week/month**	**Wk -4 to Day 0**	**Wk 0**	**Wk 1**	**Wk 2**	**Wk 3**	**Wk 4**	**Wk 8**	**Wk 12/Mth 3**	**Mth 6**	**Mth 9**	**Mth 12**	**Mth 15**	**Mth 18/Wk 77**	**Withdrawal**
Informed consent	X													
Registration/Demographics	X													
SVC	X													
ALS History	X													
ALS Interventions	X	x						x	x	x	x	x	x	x
Medical History	X													
Neurological Exam^1^	X													
Inclusion/Exclusion Criteria	X	x												
Physical Exam (inc vital signs)	x	x						x	x	x	x	x	x	x
ALSFRS-R		x						x	x	x	x	x	x	x
EQ-5D		x						x	x	x	x	x	x	x
HADS		x						x	x	x	x	x	x	x
ECG	x								x		x		x	
Haematology	x					x	x	x	x	x	x	x	x	
Biochemistry	x		x	x	x	x	x	x	x	x	x	x	x	
Dose Monitoring (Li levels)	x^3^	x	x	x	x	x	x	x	x	x	x	x	x	x
Urine pregnancy Test^2^	x	x				x	x	x	x	x	x	x	x	x
Thyroid Function Tests	x								x		x		x	
Concomitant Medications	x	x	x	x	x	x	x	x	x	x	x	x	x	x
Adverse Events Form		x	x	x	x	x	x	x	x	x	x	x	x	x
Trial Medication Log		x				x	x	x	x	x	x	x	x	x
Drug Dispensing & Returns		x				x	x	x	x	x	x	x	x^4^	x ^4^
Patient Medication Guess													x	x
Physician Medication Guess													x	x

Withdrawal Form														x

Note: where an "x" is contained within a field this denotes that the associated data will be collected at the identified time point.

1 The neurological exam must be performed by a physician

2. Women of child-bearing potential only

3. Can be completed at anytime during the screening window but must be completed before patient is randomised

4. Returns only not dispensing

##### Statistical Considerations

The Statistical Analysis Plan will be written and signed off by the TSC and DMEC in advance of database lock. Data analysis will be performed by the study statistician at Newcastle Clinical Trials Unit, using a password-protected computer in a private office.

##### Statistical Analysis

Sensitivity analyses will include per-protocol and per-treatment analyses, as specified in the Statistical Analysis Plan to be developed by the Trial Statistician and approved by the TSC and the DMEC prior to database lock.

No interim analysis is planned although pre-defined stopping criteria will be discussed by the Trial Steering Committee (TSC) and the Independent Data Monitoring and Ethics Committee (DMEC) and agreed if appropriate.

The population used in the efficacy analyses is the Intention to Treat (ITT) population. This population will consist of all patients randomised to treatment, who took at least one dose of double-blind treatment, regardless of compliance with the study protocol and procedures. If the primary analysis event (i.e., death) does not occur, a patient's data will be considered censored either on his/her planned study end date, or on the cut-off date for the study as a whole, or when the patient becomes lost to follow-up, whichever is the earlier. Data will be included in the analysis regardless of whether the patient is still receiving double-blind study treatment or complying with the prescribed regimen. The Trial Steering Committee and/or Data Monitoring Committee may decide that sensitivity analyses (endpoints and population to be defined) are also required. The population used to assess safety and tolerability will consist of all patients randomised to treatment that took at least one dose of double-blind treatment and provided at least some data thereafter, regardless of compliance with the study protocol and procedures. Descriptive statistics of baseline data and summaries of safety and tolerance data will be further subdivided by randomisation stratum (site of onset of the disease, centre).

Demographics and population characteristics: Deviations from the protocol, with regard to both the entry criteria and the scheduled assessments and examinations, will be summarized. Individual deviations will be detailed and (if appropriate) commented upon. Treatment assignment by stratum and study site, and the numbers of measurements available for each parameter will be presented. Demographics, medical history, history of the disease, scores at baseline on all variables and scales assessed, and medication and other treatment received on entry will be summarized with tabular presentation of baseline comparability.

##### Efficacy

#### Primary analysis

The primary outcome is survival at 18 months. Survival rates at 18 months in the two arms (Lithium treatment versus control) will be compared using a Kaplan-Meier analysis and log rank test. Results will also be given in the form of a 95% confidence interval for the relative risk of survival.

#### Secondary analysis

Secondary outcomes including the ALSFRS-R will be assessed at baseline, 3, 6, 9, 12, 15, and 18 months. The main statistical challenge in analysing such data is to take into account incomplete data which will be due to a number of factors most notably of which will be the death of individual patients; in this study we anticipate that survival rates may differ by at least 20%. It is necessary to take into account the differing drop out rates and the non-randomness of the drop out when comparing functional status between the two groups. This will be done by jointly modelling the survival data and repeated measurements data [33]. The survival data are analysed using a Cox proportion hazards model incorporating random effects. Functional outcomes are modelled using mixed models appropriate for repeated measures. A key feature of each of these models is that within each of them it is possible to fit a latent variable that can be conceptualised as the patient's propensity to experience poor outcomes (in the context of survival analysis this is usually referred to as frailty). It is the inclusion of this latent variable that allows us to adjust our estimates of the treatment effect to allow for the different rates of drop out in each group. Both models are estimated simultaneously; parameter estimates are based on maximising the joint likelihood over both the survival and repeated measures data. Within this framework we will estimate the effect of Lithium treatment on survival and functional status/quality of life allowing for key baseline covariates including site of onset of disease and possible differences between centres. These methods will be implemented using software that has been developed as part of an MRC funded programme of work (Grant G0400615; statistical methodology for longitudinal studies in clinical research; Williamson PR, Diggle PJ and Henderson R, unpublished work).

#### Sample Size Calculation

The sample size is based on detecting a difference in survival rates at 18 months using Fleiss' method for a proportion incorporating a continuity correction. Two groups of 110 subjects will give us 80% power to detect a difference of 17.5% in survival rates (65% versus 82.5%) assuming a type 1 error rate of 5%. It is anticipated that we will have complete survival data on all subjects recruited. The parameters included in this calculation were informed by the database of Professor V Meininger (Hôpital PitiéSalpétrière, Paris). The hypothesised difference in survival rates of 17.5% is clearly clinically very significant but less than that which might be expected on the basis of previous work [5]. Sample size was calculated defining survival as death alone (i.e., not including tracheostomy or Permanent Assisted Ventilation, PAV). Loss of follow-up per year for the primary outcome measure was set at 0%-in previous studies we have been able to ascertain survival for all subjects who have been recruited. In the most recent multicentre European trial, the unadjusted survival rate for patients with similar inclusion criteria was ~60% on riluzole alone (V Meininger, personal communication, unpublished data). For functional measures, databases from recent large-scale trials have indicated that the average slope of decrease in ALSFRS-R score on riluzole plus placebo is approximately -10 points per year with a variation coefficient ranging from 1.2 to 1.5 (with SD ranging from 12 to 15 for ALSFRS-R) [39,40]. Assuming the survival rates stated above our proposed sample size will give us 88% power to detect an effect size (or standardised difference) of 0.5 in our functional measures.

#### Recruitment

Patient recruitment began in June 2009 and the last patient is expected to complete the trial protocol in November 2011.

## Discussion

This protocol will allow us to determine if lithium carbonate is effective in ALS as suggested by an earlier study [5]. Patient recruitment began in June 2009 and the last patient is expected to complete the trial protocol in November 2011.

## Abbreviations

MND: Motor neuron disease; ALS: Amyotrophic lateral sclerosis; MHRA: Medicines and Health Regulatory Authority; NICE: National Institute for Clinical and Health Excellence; MRC: Medical Research Council; MMT: Mini-mental test; ALSFRS-R: ALS Functional Rating Scale-Revised; FVC: Forced vital capacity; QoL: Quality of life; DeNDRoN: Dementias and Neurodegenerative Diseases Research Network; SOP: Standard Operating Procedure; BNF: British National Formulary; ULN: Upper limit of normal; ALT: Alanine transaminase; SVC: Slow vital capacity; EMG: Electromyography; HADS: Hospital anxiety and depression scale; EQ-5D: EQ-5D quality of life scale; SAE: Serious adverse event; PI: Principal Investigator; GP: General Practitioner; GMP: Good Manufacturing Practice; GCP: Good Clinical Practice; ACE: Angiotensin converting enzyme; eCRF: electronic Clinical Record File; SSRI: Selective Serotonin Reuptake Inhibitor; PCV: Packed cell volume; MCV: Mean corpuscular volume; MCH: Mean corpuscular haemoglobin; MCHC: Mean corpuscular haemoglobin concentration; MH&N CTU: Mental Health and Neurosciences Clinical Trials Unit; EC: European Commission; KCL: King's College London; UKCRN: United Kingdom Clinical Research Network; TSC: Trial Steering Committee; DMEC: Data Monitoring and Ethics Committee; ITT: Intention to treat; PAV: Permanent assisted ventilation.

## Competing interests

The authors declare that they have no competing interests.

## Authors' contributions

AAC is the current Chief Investigator. PJS, CAY and KEM are PIs for the trial and contributed to the protocol. MT is the LiCALS Trial Manager and contributed to the protocol. JK is the LiCALS Data Manager and contributed to the protocol. CLM is the Manager of the CTU involved in the trial, supervises the trial management and data management and contributed to the protocol. INS is the Trial Statistician and contributed to the protocol. PNL was the original Chief Investigator and wrote the protocol. All authors except KEM sit on the Trial Management Group. All authors contributed to the manuscript. All authors read and approved the final manuscript.

## UKMND-LiCALS members

Ambily Sathish, Research Nurse, Manchester

Amina Chaouch, Blinded Physician, Preston

Ammar Al-Chalabi, Chief Investigator, King's College Hospital, London

Amy Palmer, Trial Administrator, Plymouth

Andrea Stutt, Research Nurse, Newcastle

Andrew Dougherty, Research Nurse, King's College Hospital, London

Ann Cochrane, Clinical Research Officer, NHNN, London

Annette Taylor, Research Nurse, Sheffield

Biruk Asfaw, Research Nurse, Birmingham

C Oliver Hanemann, Principal Investigator, Plymouth

Carlos Guevara, Unblinded Physician, King's College Hospital, London

Carolyn Young, Principal Investigator, Liverpool

Catherine Whatley, Unblinded Research Nurse, Oxford

Cathy Ellis, Unblinded Physician, King's College Hospital, London

Ceryl Harwood, Unblinded Physician, Sheffield

Channa Hewamadduma, Blinded Sub-investigator, Sheffield

Chinea Eziefula, Clinical Officer, NHNN, London

Chris Murphy, Unblinded Physician, Manchester

Christine Cosby, Clinical Trial Unit Manager, Plymouth

Christopher McDermott, Unblinded Physician, Sheffield

Claire McHugh, Blinded Physician, Preston

Claire Merritt, Research Nurse, Oxford

Clare Williams, Unblinded Research Nurse, Oxford

David Paling, Blinded Physician, Preston

Dave Watling, Clinical Trial Unit Manager, Liverpool

David Burn, Unblinded Physician, Institute for Ageing and Health, Newcastle

David Rog, Unblinded Physician, Manchester

Dominic Sexton, Research Nurse, Manchester

Douglas Mitchell, Principal Investigator, Preston

Elizabeth Johnson, Research Nurse, Manchester

Emma Oughton, Research Nurse, Manchester

Erica Waines, Trial Administrator, Sheffield

Faye OKeeffe, Trial Administrator, Manchester

Fiona Evans, Unblinded Physician, Liverpool

Gemma Woods, Unblinded Research Nurse, Manchester

Gill Mill, Research Nurse, Sheffield

Gill Siuda, Research Nurse, Oxford

Hannah Hollinger, Research Nurse, Sheffield

Hisham HM Hamdalla, Blinded Co-investigator, Manchester

Helen Beaumont-Kellner, Research Nurse, Manchester

Helen Vanek, Unblinded Research Nurse, Manchester

Hugh Rickards, Unblinded Physician, Birmingham

Ibrahim Imam, Unblinded Physician, Plymouth

Iracema Leroi, Unblinded Physician, Manchester/Preston

Jan Clarke, Research Nurse, NHNN, London

Jane Houghton, Research Nurse, Newcastle

Janice Birt, Unblinded Research Nurse, Preston

Janiki Panicker, Unblinded Physician, Liverpool

Jennifer Smith, Trial Administrator, Manchester

Joanna Glennon, Unblinded Research Nurse, Oxford

Joanne Brown, Research Nurse, Newcastle

John Ealing, Principal Investigator, Manchester

Jonathan Anderson, Research Nurse, NHNN, London

Jonathan Williams, Blinded Sub-investigator, Oxford

Justine Adams, Trial Administrator, Oxford

Karen Morrison, Principal Investigator, Birmingham

Kate O'Hanlon, Unblinded Research Nurse, Liverpool

Katie Sidle, Unblinded Physician, NHNN, London

Kevin Talbot, Principal Investigator, Oxford

Leanne Walker, Clinical Officer, Plymouth

Lindsey Copeland, Trial Administrator, Manchester

Lokesh Wijesekera, Unblinded Physician, King's College Hospital, London

Louise Pate, Research Nurse, Liverpool

Lucy Partington-Jones, Unblinded Research Nurse, Manchester

Lynne Savage, Research Nurse, Birmingham

Lynne Wyatt, Research Nurse, Liverpool

Marianne Hare, Unblinded Research Nurse, Preston

Martin R Turner, Unblinded Physician, Oxford

Mary Jo Trimmer, Research Nurse, Plymouth

Meneka Sidhu, Blinded Physician, Preston

Michael Grieves, Trial Administrator, Newcastle

Muhammad Rafiq, Blinded Sub-investigator, Sheffield

Nazir Sharaf, Unblinded Physician, Preston

Nichola Ritchie, Unblinded Research Nurse, Manchester/Preston

Nick Davies, Unblinded Physician, Birmingham

Nicola Maycock, Clinical Research Officer, NHNN, London

Nigel Leigh, Previous Chief Investigator, King's College Hospital, London

Nikolay Dimitrov, Blinded Physician, King's College Hospital, London

Pamela Shaw, Principal Investigator, Sheffield

Partha Ray, Unblinded Physician, Liverpool

Paula Nuttall, Unblinded Research Nurse, Preston

Phil Paterson, Research Nurse, Manchester

Rachael Hibberd, Research Nurse, Sheffield

Rachel Hornabrook, Research Nurse, Birmingham

Rehiana Ali, Blinded Sub-investigator, Liverpool

Reshma Shah, Clinical Officer, NHNN, London

Reza Sadjadi, Unblinded Physician, King's College Hospital, London

Richard Orrell, Principal Investigator, NHNN, London

Richard Sylvester, Unblinded Physician, NHNN, London

Robert Addison-Jones, Research Nurse, Preston

Robin Howard, Unblinded Physician, NHNN, London

Roisin Turner, Research Nurse, Preston

Rupert Mcshane, Unblinded Physician, Oxford

Saiffuddin Shaik, Unblinded Physician, Preston

Samirah Anane, Blinded Physician, Preston

Sarah Dhariwal, Research Nurse, Birmingham

Steven Dodds, Unblinded Research Nurse, Newcastle

Sue Palmer, Research Nurse, Birmingham

Susie Crawford, Research Nurse, Sheffield

Syed Zaidi, Blinded Physician, Preston

Tahir Majeed, Principal Investigator, Preston

Theresa McCarthy, Research Nurse, Birmingham

Theresa Walsh, Research Nurse, Sheffield

Tien Khoo, Unblinded Physician, Institute for Ageing and Health, Newcastle

Tim Williams, Principal Investigator, Newcastle

Val Russell, Research Nurse, Oxford

Wendy Barrett, Research Nurse, Oxford

NHNN is the National Hospital for Neurology and Neurosurgery

## Pre-publication history

The pre-publication history for this paper can be accessed here:

http://www.biomedcentral.com/1471-2377/11/111/prepub

## Supplementary Material

Additional file 1 **Appendix for Protocol for a double-blind randomised placebo-controlled trial of Lithium Carbonate in patients with Amyotrophic Lateral Sclerosis (LiCALS) [EudraCT number: 2008-006891-31]**. More detail on methods for compliance and withdrawal rules, data monitoring, quality control and quality assurance, access to source data, pharmacovigilance, ethics and regulatory issues, finance, insurance, and the publication policy. Click here for file
