# Supplementary material for: Protocol for a double-blind randomised placebo-controlled trial of lithium carbonate in patients with amyotrophic Lateral Sclerosis (LiCALS) [Eudract number: 2008-006891-31]
Source: BMC Neurol. 2011 Sep 21;11:111. doi: 10.1186/1471-2377-11-111 (PMC3189869; doi:10.1186/1471-2377-11-111)
Supplement: Additional file 1 — Appendix for Protocol for a double-blind randomised placebo-controlled trial of Lithium Carbonate in patients with Amyotrophic Lateral Sclerosis (LiCALS) [EudraCT number: 2008-006891-31]. More detail on methods for compliance and withdrawal rules, data monitoring, quality control and quality assurance, access to source data, pharmacovigilance, ethics and regulatory issues, finance, insurance, and the publication policy. [file 1471-2377-11-111-S1.DOC]

**Appendix for Protocol for a double-blind randomised placebo-controlled trial of Lithium Carbonate in patients with Amyotrophic Lateral Sclerosis (LiCALS) [EudraCT number: 2008-006891-31]**

**Compliance and Withdrawal**

**Subject Compliance**

Where feasible, study visits will coincide with routine clinical follow-up, with good compliance expected. Visit windows should ensure good visit attendance; non-attendance for study visits will prompt follow-up by telephone.

IMP compliance will be assessed by checking and recording the returned number of tablets returned at each participants visit. Study drug accountability will be assessed and documented by local pharmacy. Experience with recent large international trials, comprising >3000 patients, shows that compliance with study drug is high (>80% compliance in 80% of subjects). In the North American minocycline trial compliance was >90% with active drug and in other recent trials compliance has ranged between 80-90 at 18 months [39, 40]. The Italian group claims (personal communication with Dr Fornai) that patients tolerated lithium well and that there were no serious side effects.

As in other recent large survival-based studies, [39, 40] it is expected there will be no or limited loss to follow up for the primary outcome measure (death). Following relevant approvals, primary outcome data will be obtained through clinic, hospital and GP notes and verification by copies of death certificates. It is also intended that all trial participants are tracked through the Medical Research Information Service (MRIS).

**Withdrawal of Participants**

Study drug must be discontinued if: the participant decides they no longer wish to continue; recommended by the Investigator or another clinician (e.g. intercurrent illness during course of study, side effects from study drug); or the trial is terminated at the request of the DMEC.

Participants have the right to withdraw from the study at any time and for any reason, without providing a reason. The investigator also has the right to withdraw participants from the study in the event of inter-current illness, AEs, SAEs, SUSARs, protocol violations or other reasons. Should a participant decide to withdraw from the study, they will be asked to volunteer a reason for withdrawal but are at liberty not to state a reason.

Should a participant withdraw from study drug only, efforts will be made to continue to obtain follow-up data, with the permission of the patient. Subjects who withdraw from treatment early will be encouraged to return to the study site for early termination assessments, and those who terminate early will continue to have follow-up telephone calls for survival and ALSFRS-R up until week 77, providing that consent is not withdrawn.

**Data Monitoring, Quality Control and Quality Assurance:**

**Discontinuation rules**

The trial may be prematurely discontinued on the basis of predefined stopping criteria, or for other reasons given by the independent Data Monitoring & Ethics Committee, study sponsor, regulatory authority or ethics committee concerned.

**Monitoring, Quality Control and Assurance**

LiCALS is a collaboration with the Dementias and Neurodegenerative Disease Research Network (DeNDRoN) within the UK Clinical Research Network (UKCRN). The protocol has been developed through the ALS/MND Clinical Studies Group (CSG) with extensive review by clinicians, statisticians, and lay members of the CSG.

The LiCALS Trial Coordination Centre will be based in the Trials Centre of the Department of Clinical Neuroscience at the Institute of Psychiatry, King’s College London (IOP/KCL), in collaboration with the MH&N Clinical Trials Unit (MH&N CTU). Day to day management of LiCALS will be the responsibility of the Chief Investigator and the Trial Manager. The Coordinating Centre will arrange meetings of the Trial Management Group (TMG) and Trial Steering Committee (TSC) and will coordinate independent Data Monitoring and Ethics Committee (DMEC) meetings.

The TMG will comprise of the Chief Investigator (Chair), the Trial Manager, the Data Manager, Professor Pamela Shaw, Trial Statistician, the director of Newcastle Clinical Trials Unit, the MH&N Clinical Trials Unit Manager and the DeNDRoN co-ordinator. The TMG will arrange telephone conferences and provide a weekly recruitment email updates during recruitment and status reports 6-monthly thereafter. The TMG will organise a meeting for all PIs (and for key staff working on the study) to sign the protocol, and to agree the content and undergo training on SOPs, before the start of recruitment. A second investigators meeting will be held at the end of the study to review the results. The TMG will also meet face-to-face every 3 months in London or Sheffield alternately. The TMG will report to the Trial Steering Committee (TSC). All PIs will be kept informed of TSC and DMEC advice and consulted by e-mail or teleconference as required.

The TSC will consist of: an independent chair; the trial statistician; an independent neurologist; a representative of the MND Association; the Chief Investigator and one other LiCALS trial neurologist. The TSC will be responsible for approving the trial protocol, and overseeing the conduct of the study, including advising on continuing or stopping the study in the light of advice form the DMEC. Meetings of the TSC will be at least annual.

Membership of the DMEC will comprise: an Independent Chair; an independent MND neurologist; and an independent statistician. The DMEC will have access to the unblinded data and will monitor the progress of the trial in terms of safety and ethical issues. The DMEC may advise the TSC to continue or to stop the trial according to pre-agreed stopping rules.

The Principal Investigators will be responsible for the day-to-day study conduct at site. This includes: establishing and carrying out the trial at his/her centre, in accordance with international, national and local law and regulations and GCP; ensuring that all site specific documentation is complete and correct, and that all staff involved in the trial are compliant with Trust, GMC and other relevant regulations, are appropriately trained in those aspects of GCP relevant to their role in the study and are familiar with the trial protocol; managing recruitment on target and collecting and submitting accrual and outcome data in a timely manner; responding in timely fashion to requests from the Trial Coordinating Centre for information; providing and responding promptly to SAEs and SUSAR reports; agreeing to monitoring and audit visits as required.

Quality control will be maintained through adherence to KCL (IoP) standard operating procedures (SOPs), and relevant MH&N CTU SOPs, study protocol, the principles of GCP, research governance and regulatory requirements.

Data management will be supervised by the study statistician, the study data manager, the Director of the Newcastle Clinical Trials Unit (NCTU), and by the MH&N Clinical Trials Unit Manager using the MH&N CTU InferMed MACRO database.

Statistical analysis will be carried out by the study statistician at the NCTU.

Central and site monitoring of study conduct and data collected will be performed by the Trial Manager in collaboration with the MH&N CTU on behalf of the Sponsor. The main areas of focus will include consent, serious adverse events, essential documents, primary and secondary outcome measures and drug accountability & management. All monitoring findings will be reported and followed up with appropriate persons in a timely manner.

The study may also be subject to audit or inspection by KCL under their remit as sponsor, or by the MHRA or other regulatory bodies to ensure adherence to GCP and regulatory requirements.

**Direct access to source data and documents**

The investigators agree to provide full access to all source data, study data and materials to the trust research governance department, ethics committee, regulatory authority and trial manager for purposes of monitoring, audit or inspection.

**Pharmacovigilance**

**Definitions:**

***Adverse event (AE):*** Any untoward medical occurrence which does not necessarily have a causal relationship with the treatment. “Treatment” includes all investigational and non-investigational agents administered during the course of the study. Medical conditions/diseases present before starting study treatment are only considered adverse events if they worsen after starting study treatment.

***Adverse Reaction (AR):*** Any untoward or unintended responses to an Investigational Medicinal Product (IMP) related to any dose administered - All AEs judged by either the reporting investigator or the sponsor as having a reasonable suspected causal relationship to a medicinal product (i.e. definitely, probably or possibly related) qualify as adverse reactions. The expression “reasonable causal relationship” means to convey in general that there is evidence or argument to suggest a causal relationship.

**Causality:**

The assignment of the causality should be made by the investigator responsible for the care of the participant using the definitions in Table 4 below. If any doubt about the causality exists, the investigator should inform the Chief Investigator. In the case of discrepant views on causality between the investigator and others, all parties will discuss the case. In the event that no agreement is made, the MHRA, main REC and other bodies will be informed of both points of view.

**Table 4**

| Relationship | Description |
| --- | --- |
| None | There is no evidence of any causal relationship to study treatment. |
| Remote | There is little evidence to suggest there is a causal relationship (e.g. the event did not occur within a reasonable time after administration of the trial medication). There is another reasonable explanation for the event (e.g. the participant’s clinical condition, other concomitant treatment). |
| Possible* | There is some evidence to suggest a causal relationship (e.g. because the event occurs within a reasonable time after administration of the trial medication). However, the influence of other factors may have contributed to the event (e.g. the participant’s clinical condition, other concomitant treatments). |
| Probable* | There is evidence to suggest a causal relationship and the influence of other factors is unlikely. |
| Definite* | There is clear evidence to suggest a causal relationship and other possible contributing factors can be ruled out. |

* Reportable to MHRA

***Serious Adverse Event (SAE) or Serious Adverse Reaction (SAR):*** any untoward medical occurrence or effect that at any dose results in death, is life-threatening (refers to an event in which the subject was at risk of death at the time of the event; it does not refer to an event which hypothetically might have caused death if it were more severe), requires hospitalisation, or prolongation of existing inpatient hospitalisation, results in persistent or significant disability or incapacity, is a congenital anomaly or birth defect. Medical judgement should be exercised in deciding whether an AE/AR is serious in other situations. Important AE/ARs that are not immediately life-threatening or do not result in death or hospitalisation but may jeopardise the subject or may require intervention to prevent one of the other outcomes listed in the definition above, should also be considered serious.

***Suspected, Unexpected Serious Adverse Reaction (SUSAR):*** an adverse reaction that is both unexpected and serious. An adverse reaction is ‘unexpected’ if its nature or severity is not consistent with the applicable product information.

**Expected adverse reactions:**

Most adverse drug reactions that occur in this study, whether serious or not, will be expected treatment-related toxicities as lithium carbonate (LiCO_3_) has a well-established side effect profile. LiCO_3_ can cause renal impairment (particularly impaired urinary concentration and polyuria), hypothyroidism, leukocytosis, tremor, gastrointestinal upset, polydipsia, hyperparathyroidism, hypercalcaemia, weight gain and oedema. Lithium can also cause neurotoxicity, which can happen without an increase in blood lithium levels. Neurotoxicity usually follows interaction with medications: Drugs implicated include, but are not limited to, selective serotonin reuptake inhibitors (SSRI’s), calcium channel blockers, carbamazepine, phenytoin, neuroleptics and methyldopa.

Signs of intoxication are blurred vision, increasing gastro-intestinal disturbances (anorexia, vomiting, diarrhoea), muscle weakness, increased CNS disturbances (mild drowsiness and sluggishness increasing to giddiness with ataxia, coarse tremor, lack of co-ordination, dysarthria), and require withdrawal of treatment; with severe overdosage (serum-lithium concentration above 2 mmol/litre) hyperreflexia and hyperextension of limbs, convulsions, toxic psychoses, syncope, renal failure, circulatory failure, coma, and occasionally, death; goitre, raised antidiuretic hormone concentration, hypothyroidism, hypokalaemia, ECG changes, and kidney changes may also occur.

The adverse events reported above are expected, in the sense that they are possible known side-effects of the study medication, and therefore do not require expedited reporting to the regulatory authorities and ethics.

**Protocol Specifications**

For purposes of this protocol any serious adverse events will be recorded throughout the duration of the trial until 30 days after cessation of study drug, or until resolution.

Non-serious adverse events will be recorded throughout duration of trial until 30 days after cessation of study drug. Serious adverse events exclude any pre-planned hospitalisations not associated with clinical deterioration.

**Recording & Reporting Serious Adverse Events or Reactions**

All adverse events and all serious adverse events should be recorded. Depending on the nature of the event, the reporting procedures below should be followed. Any questions concerning adverse event recording/reporting should be directed to the Chief Investigator in the first instance. A flowchart (figure 1) is given below to aid in the recording/reporting procedures.

***Non-serious Adverse Events:*** All non-serious adverse events will be recorded on the study CRF. Severity of all AEs will be graded on a three-point scale of intensity (mild, moderate, severe):

***Mild:*** Discomfort is noticed, but there is no disruption of normal daily activities.

***Moderate:*** Discomfort is sufficient to reduce or affect normal daily activities.

***Severe:***  Discomfort is incapacitating, with inability to work or to perform normal daily activities.

Relation of an AE to treatment should be assessed by the investigator/delegate (must be a clinician) at site, using Table 4.

Investigators will be responsible for managing all adverse reactions according to local protocols as study drug is licensed for use in other indications.

***Serious Adverse Event / Reaction (SAE/SAR, including SUSARs):*** All SAEs, SARs and SUSARs shall be recorded and reported on the serious adverse event form to the Chief Investigator / delegate within 24 hours of learning of its occurrence. The initial report can be made by completing the serious adverse event form, and faxing to the MH&N CTU (020 7848 5229). A record of this notification (including date of notification) must be clearly documented to provide an audit trail. In the case of incomplete information at the time of initial reporting, all appropriate information should be provided as follow-up as soon as this becomes available.

Relationship of the SAE to the treatment should be assessed by the investigator/delegate (must be a clinician) at site, as should the expected or unexpected nature of any serious adverse reactions. As this is a blinded trial involving a placebo and active drug, seriousness, causality and expectedness should be evaluated as though the patient was on active drug.

SUSAR (Suspected Unexpected Serious Adverse Reaction) reporting responsibility to the MHRA will be held by the Joint Clinical Trials Offices (JCTO) at the KCL. The JCTO will report SUSARs and other SARs (Serious Adverse Reaction) to the regulatory authorities (MHRA, competent authorities of other EEA (European Economic Area) states in which the trial is taking place.

The Chief Investigator will report to the relevant ethics committees. Reporting timelines are as follows: SUSARs which are fatal or life-threatening must be reported not later than 7 days after the sponsor is first aware of the reaction. Any additional relevant information must be reported within a further 8 days. SUSARs that are not fatal or life-threatening must be reported within 15 days of the sponsor first becoming aware of the reaction.

The Chief Investigator will provide an annual report of all SARs (expected and unexpected), and SAEs which will be distributed to the Sponsor (JCTO), MHRA and the REC.

As this is a blinded study, cases that are considered SUSARs would have to be unblinded to the MH&N CTU manager prior to reporting to the JCTO and main REC. This is to be done on behalf of the sponsor by the unblinded “safety monitoring” physician at each site. Only those events occurring among patients on the active drug (unless thought to be due to the excipient in the placebo) should be considered SUSARs.

All investigators will be informed of all SUSARs occurring throughout the study on a case-by-case basis. This will be regardless of medication administered in order to avoid the risk of inadvertently unblinding investigators, unless this information is needed for medical management of patients.

The Chief Investigator will ensure KCL (IOP) as sponsor is notified of any SUSARs.

Adverse Event

Non-serious

Serious

PI assesses relationship to study drug

Complete CRF

Unrelated to study drug

Related to study drug

PI assesses expectedness

Report to MH&N CTU and Sponsor and complete CRF

**Figure 1**

* The Chief Investigator / Trial coordinating centre MUST be informed of all SAEs or SUSARs within 24 hours of learning of its occurrence. A record of this notification (including date of notification) must be clearly documented to provide an audit trail.

***Contact details for reporting SAEs and SUSARs:***

Trial Manager, MH&N CTU: Fax 020 7848 5229

***Pregnancy***

Should a trial participant become pregnant during the trial, she will be immediately withdrawn from study treatment, and the pregnancy followed up until outcome. The need to unblind will be considered on a case by case basis.

**Ethics & Regulatory Issues**

The conduct of this study will be in accordance with the recommendations for physicians involved in research on human subjects adopted by the 18th World Medical Assembly, Helsinki 1964 and later revisions.

Information sheets will be provided to all eligible subjects and written informed consent obtained prior to any study procedures. Participants will be provided with a copy of the completed consent form for their records.

Favourable ethical opinion and Clinical Trial Authorisation will be sought prior to commencement of the study. Local approvals will also be sought, including site specific assessment and trust R&D approval. The participating site must also sign a Clinical Trial Agreement (CTAg) with the study sponsor. The Trial Coordination Centre will require a written copy of local approval documentation and a copy of the signed CTAg before initiating each centre and accepting participants into the study.

**Finance and Insurance**

The Motor Neurone Disease Association is funding this study and the NIHR Dementia and Neurodegenerative Diseases Clinical Research Network have provided service support costs.

The participating NHS Trusts have liability for clinical negligence that harms individuals towards whom they have a duty of care. NHS indemnity covers NHS staff and medical academic staff with honorary contracts conducting the trial. University insurance covers research staff that have their substantive contract with the University for potential liability arising from negligence in study design. There are no arrangements for non-negligent compensation.

**Publication Policy**

The data will be the property of the Sponsor. Publication will be the responsibility of the Chief Investigator. Results from the study will be submitted for publication by the investigators only, in international medical journals. All manuscripts, abstracts or other modes of presentation will be reviewed by the TSC and DMEC prior to submission. No reference will be made to any particular study subject. Results of the study will also be reported to the Sponsor and Funder in the required format.

Participants will be informed about their treatment and their contribution to the study at the end of the study, including a summary of the results.
